# Taking Exception. Reduced mortality leads to population growth: an inconvenient truth

**DOI:** 10.9745/GHSP-D-14-00062

**Published:** 2014-05-13

**Authors:** James D Shelton

## Abstract

Reduced mortality has been the predominant cause of the marked global population growth over the last 3/4 of a century. While improved child survival increases motivation to reduce fertility, it comes too little and too late to forestall substantial population growth. And, beyond motivation, couples need effective *means* to control their fertility. It is an inconvenient truth that reducing child mortality contributes considerably to the population growth destined to compromise the quality of life of many, particularly in sub-Saharan Africa. Vigorous child survival programming is of course imperative. Wide access to voluntary family planning can help mitigate that growth and provide many other benefits.

The 2014 Gates annual letter, “3 Myths That Block Progress for the Poor,” makes many valid points about development, and, commendably, it strongly supports family planning.^1^ However, in arguing against what it termed a “myth”—that saving lives leads to overpopulation—ironically, it succumbs to a common misunderstanding about reduced mortality and population growth.

## THE CHILD SURVIVAL HYPOTHESIS

The letter's basic proposition is: “When children survive in greater numbers, parents decide to have smaller families.” The inference is that reduced child mortality will somewhat automatically produce a corresponding and largely compensatory reduction in fertility levels, with little appreciable overall impact on population growth.

This concept, sometimes termed “the child survival hypothesis,” was discussed and researched considerably, particularly during the 1970s. It has some intuitive credence and demographic support, because often historically when death rates began to fall, declines in birth rates followed.^2^ However, such an association does not prove causality. Indeed, historically sometimes the 2 rates have declined fairly concurrently, and there are many examples where birth rates began to fall before death rates.^3^ Notably, the very intensive province-by-province “Decline of Fertility in Europe” analysis found that while there was some weak association between child mortality and fertility decline, fertility decline was also somewhat associated with industrialization, urbanization, literacy, and women's employment.^3^ But the study's overriding finding was that fertility declines spread rapidly “like an epidemic” through provinces that shared a cultural as well as spatial location, supporting strong ideational and normative explanations—that is, that people recognized that limiting family size was both feasible and acceptable to do.

Fertility declines spread rapidly through European communities that shared cultural and spatial location.

**FIGURE 1.**
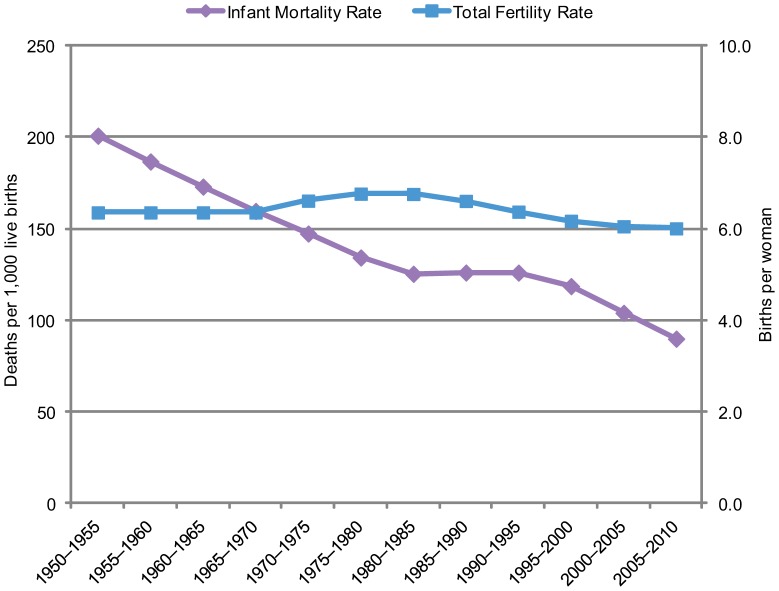
Infant Mortality and Total Fertility, Nigeria, 1950–2010

Of course, the situation among modern developing countries varies and is different from that in Europe a century ago. For one thing, child mortality rates have typically declined much more rapidly in developing countries. And modern communications have fueled rising aspirations for many. But notably, substantial mortality declines in a number of countries, especially in Africa, have not yet been followed by appreciable declines in fertility. A prime example is Nigeria (Figure 1). Despite declines in infant mortality over many years, total fertility has persisted at about 6 children per woman.^4^

Declines in mortality have not yet always been followed by substantial declines in fertility.

In addition, for the child survival impetus to work, people must also perceive the decline in mortality and act on it. While the literature on that perception is limited, it suggests there is a major time lag before people do perceive such declines.^5^ Actually, in all likelihood, the major reason death and birth rates often fall over a similar time frame is due to general modernization changes in society—economic, educational, and social improvements, modern awareness, women's empowerment, rising aspirations, and better access to services that lead to declines in both mortality and fertility levels. But most importantly, for fertility levels to decline, women and couples must have good *means* to control their fertility, in addition to motivation.

Women and couples must have good means to control their fertility, in addition to motivation.

So yes, there is indeed something of a virtuous cycle in that lowered child mortality over time very likely does contribute to reduced fertility. But in and of itself, the effect is *too little and too late*.

## WHAT HAS CAUSED MODERN POPULATION GROWTH?

Predominantly declines in mortality. For most of human history, global population growth was extremely slow, because mortality and fertility levels were in fairly close equilibrium. But recent times have taken us rapidly to 7 billion and counting.^4^ As demonstrated in the classic work of Thomas McKeown, *The Modern Rise of Population,* the only plausible explanation is declines in mortality.^6^ Consider, there are only 3 possible determinants of population change—fertility, migration, and mortality. Fertility may sometimes have increased marginally but, overall, certainly not appreciably; and migration is net zero for the planet, with mostly some out-migration for most developing countries. That leaves only mortality decrease as the primary explanation for the profound increase in population.

Decreased mortality is the primary explanation for the profound increase in population over the past several decades.

Moreover, reduced *child* mortality plays a huge role. Deaths to children under 5 typically account for at least half of all deaths in pre-transition societies, and child mortality declines have been dramatic. In addition, child survival contributes to population “momentum” because most of those surviving children will eventually have children themselves. Thus, not only does reducing mortality contribute to rapid population growth, it is the *predominant* cause, notwithstanding the partial virtuous cycle that reduced child mortality may partially help over time to reduce fertility levels.

### Does Substantial Population Growth Matter?

As Malcom Potts points out in this issue,^7^ estimates from the United Nations (UN) for the global population in 2100 range from 6.8 billion to 16.6 billion. Although these are very abstract numbers, the conclusion seems inescapable that the difference in the estimates would have major impact on quality of life for the earth's inhabitants. Indeed, the recent Royal Society report, “People and the Planet,” voiced major concern about *current* shortages of water, food, and fuel as well as environmental degradation, climate change, and urbanization.^8^

**FIGURE 2.**
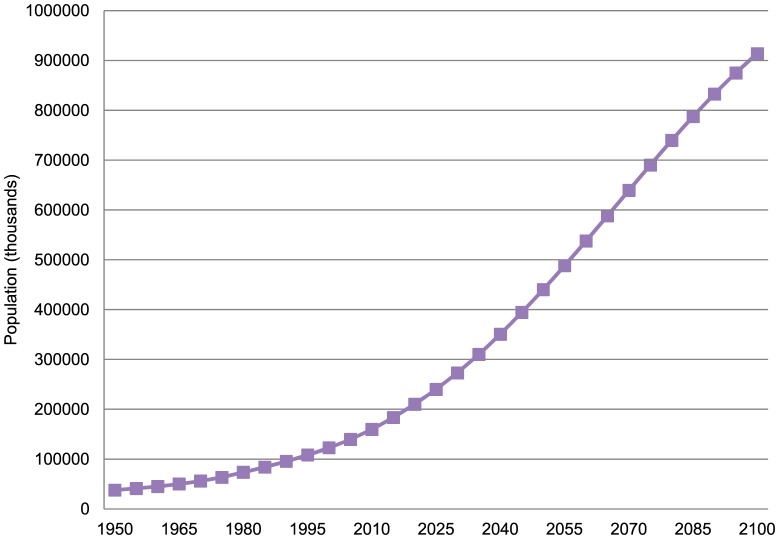
Projected Population Growth, Nigeria, 1950–2010

Population growth appears destined to affect adversely the quality of life of people in the developing world, especially Africa. A key case in point is Nigeria, where, as previously mentioned, fertility levels remain quite high despite major decreases in mortality. The ominous implications are laid out in Figure 2. According to the UN medium projection (which even assumes considerable fertility decrease), Nigeria alone will be approaching a billion people in 2100, only slightly less than the United States and all of Europe *combined*.^4^

Another even more extreme example is the environmentally fragile Sahelian country Niger. Partly because of very intensive health interventions,^9^ its infant mortality has declined to about 60/1,000 live births, but total fertility is even higher than in Nigeria, at 7.6 children per woman.^4^ Accordingly, its population is projected to increase well over 10-fold, from about 18 million today to over 200 million in 2100.^4^

Because of decreasing child mortality and fairly stable fertility levels, in both Nigeria and Niger, their population growth rates have actually been *increasing* in recent years. While the future is hard to predict, surely it must be clear that such increases in population will likely impair the quality of life severely, particularly for those most in need in these countries.

It seems it has become less fashionable to express concern about population growth in recent years, partly because such concern is somehow associated with coercive practices. However, access to family planning is itself becoming better recognized as a human right.^10^^,^^11^ While we must condemn and steadfastly guard against misguided, and I would say rare, instances of coercion, neither should we ignore the benefits, including human rights benefits, to people and the planet that can come through voluntary family planning, including slower-paced population growth.

Voluntary access to family planning is a human right.

## IMPLICATIONS OF THIS INCONVENIENT TRUTH FOR CHILD SURVIVAL AND FAMILY PLANNING PROGRAMMING

Like it or not, we face an inconvenient truth. Reducing child mortality does increase population growth, which will likely substantially impair the quality of life for those very people we wish to help. Does that mean we should curtail our child survival efforts? Not at all. We have an ethical imperative to reduce mortality, and it affirms our humanity. But in my view, it also reinforces the imperative to make a full menu of quality voluntary contraceptive services widely available, and as expeditiously as possible. Unmet need for family planning remains high in developing countries.^12^ And recent experience in Ethiopia and elsewhere demonstrates that quality family planning programming can be highly successful in advance of major socioeconomic development.^13^

We have an ethical imperative to reduce mortality, but we also have an imperative to make quality family planning services widely available.

As Potts points out and as reinforced in the Gates annual letter, the great appeal of family planning is that it has so many benefits. Those include substantial health benefits for women and children, enhanced women's empowerment, economic benefits for the family, the demographic dividend, reduced pressure on the environment, and the right to determine one's own life destiny. Not just convenient, but a compelling opportunity.
